# The association between high-arched feet, plantar pressure distribution and body posture in young women

**DOI:** 10.1038/s41598-019-53459-w

**Published:** 2019-11-20

**Authors:** Renata Woźniacka, Łukasz Oleksy, Agnieszka Jankowicz-Szymańska, Anna Mika, Renata Kielnar, Artur Stolarczyk

**Affiliations:** 1 Department of Anatomy, Faculty of Motor Rehabilitation, University of Physical Education in Krakow, Krakow, Poland; 2 Physiotherapy and Sports Centre, Rzeszow University of Technology, Rzeszow, Poland; 3 Oleksy Medical & Sports Sciences, Łańcut, Poland; 4 Institute of Health Sciences, State Higher Vocational School in Tarnow, Tarnow, Poland; 5 Department of Clinical Rehabilitation, University of Physical Education in Krakow, Krakow, Poland; 6 Institute of Physiotherapy, Medical College of Rzeszow University, Rzeszow, Poland; 7 Orthopaedic and Rehabilitation Department, Medical University of Warsaw, Poland

**Keywords:** Lifestyle modification, Risk factors

## Abstract

The aim of this study was to examine the effect of excessive feet arching (symmetrical and asymmetrical) on plantar pressure distribution and on the alignment of pelvis, spine and shoulder girdle. Eighty-one women (20–40 years old, 61 +/− 12 kg, 165 +/− 5 cm) were divided into 3 groups based on the foot arch index (Group 1 - with normally arched feet, Group 2 with one foot properly arched and the other high-arched, Group 3 with both feet high-arched). Plantar pressure distribution between the right and left foot for the forefoot, midfoot and rearfoot, respectively and body posture were assessed. A slight increase in longitudinal arch of the foot caused changes in the distribution of feet loads both between limbs and between the forefoot and rearfoot and also influenced the whole body. Asymmetrical high-arching of the feet resulted in asymmetry of lower limb load and in the height of the shoulder girdle. We have suggested that any alteration of the foot arch may be harmful to body tissues and should not be considered as correct. Due to the fact that slight increases in longitudinal arch of the foot are very common, they should be considered as a foot defect, and appropriate corrective exercises should be used to prevent forefoot overload and alterations in body posture.

## Introduction

The sedentary life style present in modern society leads to many musculoskeletal disorders in all parts of the body^1^. One of the most common are foot posture abnormalities - flat (pronating) foot and the hollow (supinating) foot^2–4^. As reported by some authors^3,5,6^, nowadays, high-arched feet are more often observed among children and adolescents than previously. This problem has been described by many authors^7–10^ but the effects of different foot posture on pelvis and spine ailments are still little known.

The foot is a part of the biokinetic chain connecting the lower limb to the spine, via the pelvis^11^. It has been suggested that the foot arch and its loading during standing and walking may affect the upper body parts^12,13^. In a normal, healthy foot, the longitudinal and transverse arches provide the most optimal foot loading and proper force distribution. However, foot arch alterations may lead to structural changes and/or affect its load distribution^14,15^. It should be noted that even slight elevations of the foot’s medial longitudinal arch above the norm may lead to changes in load and pressure distribution^16,17^. It was observed that abnormalities in the distal parts of the body may influence its proximal segments, e.g. excessive foot pronation transferred to internal rotation of the tibia, which may cause knee overloading or other changes in the proximal part of the lower limb^18–20^. Also, increased medial longitudinal arch with supination causes lower mobility of the foot^15,21^ and shock absorption mechanism weakness which predisposes to injuries^22,23^. The potential, close connection between distant body parts was also hypothesised about by Murley *et al*.^24^. They observed that the change in foot alignment by an orthosis may influence the bioelectrical activity (sEMG) of the proximal muscles in the lower limbs and the trunk^24^.

It should be noted that in some studies describing the influence of foot posture on other parts of the body, forced foot positioning was used during the experiment^8,25^. In those studies, the foot edge elevation artificially creating excessive foot arching was obtained by special platform or footwear with an insert^8,25^. But such a study protocol did not allow to observe true changes in foot load distribution because this artificial foot arching was unnatural for study participants, thus, those observations should be considered with caution. Additionally, those studies showed, that instantaneous changes in foot arch which may even create some force alterations throughout the tissues are local, and cannot affect immediately distant body parts such as the thoracic or cervical spine and/or shoulder girdle^12^. But, if excessive foot arching is permanent, the inappropriate foot posture may lead to changes in the load distribution, and therefore, through the myofascial system, abnormal tension in the soft tissues of the feet can spread to upper parts of the body^11,26^. This, in turn, can lead to the overloading of distant body parts and incorrect tension may create permanent changes in the ligament-fascial system resulting in postural alterations. It has been suggested that inappropriate tension in some parts of the body may be transmitted to distant parts of the musculoskeletal system causing overload and functional restrictions^27,28^.

Some authors have suggested that the asymmetrical joint range of motion may lead to changes in muscle and tendon length as well as function^29,30^. It was also reported that asymmetry in muscle work, strength and length are considered a strong risk factor of musculoskeletal injury^31^. Therefore, asymmetry in the arch height between feet may also be related to those ailments. We may hypothesise that excessive arching of only one foot can affect the tissues of the spine, pelvis and shoulder girdle differently than in the case of excessive arching in both feet. Deeper analysis of the influence of foot arch asymmetry may lead to more accurate treatment of musculoskeletal dysfunctions.

Many authors have reported the influence of foot posture on the lower limb joints^25,32^, on pelvis inclination^8,11^ and on the lower sections of the spine^8^. However, there is a lack of conclusive studies reporting on the impact of excessive foot arch on the proximal body parts, such as the spine and shoulder girdle. There is also a lack of studies evaluating the influence of asymmetric foot arch on changes in upper body parts. The present study has undertaken this problem for the first time.

The aim of this study was to examine the effect of excessive feet arching (symmetrical and asymmetrical) on plantar pressure distribution and on the alignment of pelvis, spine and shoulder girdle.

## Material and Methods

### Participants

**Figure 1:**
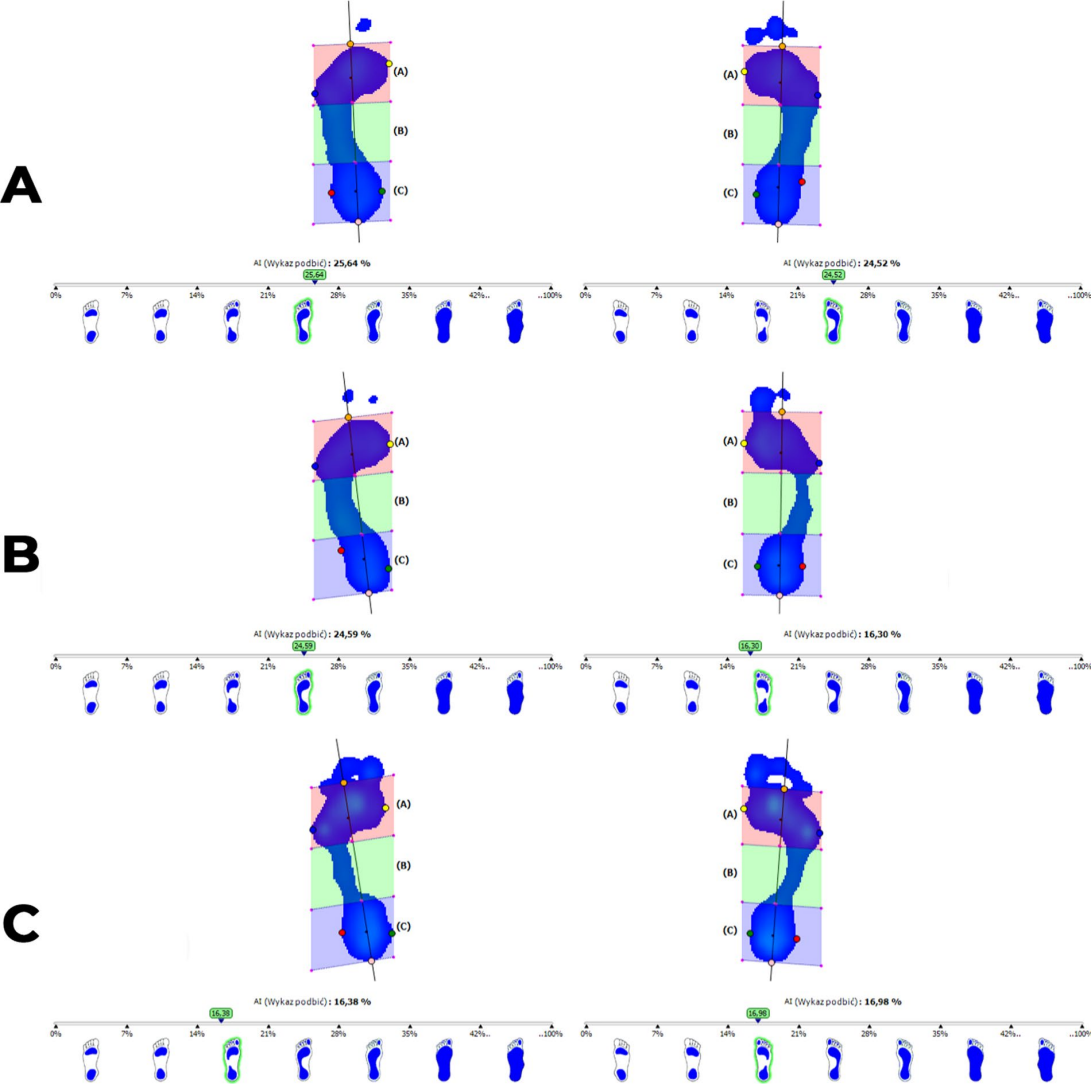
The graphical representation of pressure distribution for group V1 - women with normally arched feet (part **A** of the Figure); for group V2 - women with asymmetrically arched feet (part **B** of the Figure); group V3 – women with both high-arched feet (part **C** of the Figure).

In this study, 81 (eighty-one) women (20–40 years old, body mass 61 +/− 12 kg, body height 165 +− 5 cm) were evaluated. They were healthy and did not have any orthopaedic or neurological disorders. They were divided into 3 groups based on the measured foot arch index (AI)^9,10^ (Fig. 1).

Group 1 (V1, n = 38) - women with normally arched feet, AI = 25%+/−1.4%

Group 2 (V2, n = 23) - women with asymmetrically arched feet (one foot properly arched, AI = 23.4% +/− 1.3% and the other high-arched, AI = 15.8% +/− 1.5%)

Group 3 (V3, n = 20) - women with both feet high-arched, AI = 14% +/− 2.8%

All measurements for each woman were performed during one visit at the laboratory. The participants were informed about the research protocol in detail and gave their written informed consent to participate in the study. All procedures were performed in accordance with the 1964 Helsinki declaration and its later amendments. The approval of the Bioethics Committee at the Regional Medical Association in Krakow (No 28/KBL/OIL/2018) was obtained for this study and for whole experimental protocol.

## Procedures

### Plantar pressure distribution measurements

In each group, plantar pressure distribution was measured on the BTS P-WALK baroresistive platform (*BTS*, *Bioengineering*, *Italy*) which allowed to examine pressure distribution between the right and left foot for the forefoot, midfoot and rearfoot, respectively. Data were evaluated using G-Studio software (*BTS*, *Bioengineering*, *Italy*). Calibration of the platform was conducted prior to data collection based on the manufacturer’s instructions.

During the measurement, each evaluated woman stood barefoot, assuming an upright position in the middle of the platform, with arms to her sides. The participant was asked to stand on the platform in a relaxed, habitual position and to remain motionless for 30 seconds looking straight ahead. During the examination, the subject did not see the computer monitor displaying the right and left foot load values.

Evaluated variables:

Arch Index (%) - normal foot (18–30%), high-arched foot (<18%)

Total load (%) - the percentage of the load on the whole foot

Forefoot pressure area (%) - the percentage of the load on the forefoot

Midfoot pressure area (%) - the percentage of the load on the midfoot

Rearfoot pressure area (%) - the percentage of the load on the rearfoot

### Body posture evaluation

Body posture was evaluated using the Zebris APGMS Pointer system (*Zebris*, *Germany*). The data were evaluated using WinSpine software (*Zebris*, *Germany*). The position of the spine, pelvis and shoulder girdle was assessed.

The subject stood barefoot, in underwear, with the device placed on a tripod behind her/him, at a distance of about 80 cm, at the appropriate height, depending on the subject’s body height. The triple reference marker was attached at the body laterally, low enough not to impede probing of the spinal vertebrae and to prevent being covered by the hand or the pointer. The evaluated women stood assuming habitual posture during the whole measuring procedure, motionless and well-balanced on both feet. First, the position of the measuring device was calibrated to define its position with regard to the ground. Then, the following anatomical points were determined via the pointer device:

- shoulder points (left/right acromion)
- pelvic points (left/right spina iliaca posterior superior, left/right spina iliaca anterior superior, left/right crista iliaca).
- point T12/L1 (point between thoracic and lumbar spine)
- scapula points (left/right angulus inferior spina scapular left)

Symmetrical points are always marked starting from the left side of the body. After all points were determined, the spinal crest line was scanned three times with the tip of the pointer from C7 to S3.

## Evaluated variables

- Pelvic torsion (degrees) - the angle between the line drawn through the spina iliaca posterior superior, right and the spina iliaca anterior superior, right and the line drawn through the spina iliaca posterior superior, left and the spina iliaca anterior superior, left.
- Pelvic obliquity (degrees) - angle between the line drawn through the crista iliaca right and left, and the floor plane.
- Pelvic/shoulder obliquity (degrees) - obliquity of the acromion points in relation to the pelvis (line drawn through the crista iliaca).
- Pelvis/shoulder rotation (degrees) - rotation angle between the line drawn through the spina iliaca posterior superior left and right, and the left and right shoulder points.
- Pelvic height difference (mm) - the difference in height between the crista iliaca right and left. The point that lies lower with respect to the horizontal plane is the reference point.
- Thoracic kyphosis/Lumbar lordosis (degrees) - total angles of the thoracic or lumbar vertebral column.
- Sacral angle (degrees) - the angle between the tangent through S1 and the frontal plane.
- Total trunk inclination (degrees) - the angle between the line drawn through C7 and L5/S1, and the perpendicular.
- Scapula distance difference (mm) - the difference between the distances of the left and right shoulder blade point from the frontal plane which is defined by C7 and the two spina iliaca superior posterior points.
- Shoulder height difference (mm) - the difference in height between the right and left shoulder points (acromion). The point that lies lower with respect to the horizontal plane is the reference point.

### Statistical analysis

Statistical analysis was conducted using the STATISTICA 12.0 software package. The Shapiro-Wilk test was conducted to assess data for normality. One-way analysis of variance (ANOVA) was employed for the evaluation of significance in the differences of the measurement variables across three study groups. When a significant main effect was detected Tukey’s post-hoc test was performed. The effect size was calculated using Cohen’s *d*. Differences were considered statistically significant if the level of test probability was lower than the assumed level of significance (*p* < 0.05).

## Results

### Plantar pressure distribution (load distribution of the feet)

Total foot load (%) in a standing position varied between study group. There were no significant differences between the left and right foot in V1 and V3 groups regarding total foot load (in women with symmetrical foot arches). But, in the V2 group with asymmetric foot arches, the right foot with normal arching was significantly more loaded in relation to the left, high-arched foot (Table 1). The percentage difference was the largest and amounted to 4.7%.

**Table 1:** Comparison of plantar pressure distribution variables between study groups.

Outcome Measure	Side	Group V1	R – L^a^	Group V2	R – L^a^	Group V3	R - L^a^	V1-V2^b^	ES	V1-V3^c^	ES	V2-V3^d^	ES
Total load (%)	R	49.8 ± 3	n.s	52.3 ± 4	**p** = 0.009** **ES** = **1.17**	50.7 ± 3.5	n.s	0.007**	0.7	n.s.	0.24	n.s.	0.45
	L	50.1 ± 3		47.6 ± 4		49.3 ± 3.5		0.007**	0.7	n.s.	0.24	n.s.	0.45
Forefoot load (%)	R	43.4 ± 2.2	n.s	43.4 ± 2.2	n.s	47.8 ± 4.1	n.s.	n.s.	0.05	0.00001***	1.39	0.00001***	1.33
	L	43.2 ± 1.8		43.8 ± 5.7		47.9 ± 4.1		n.s.	0.14	0.00003***	1.48	0.001**	0.82
Midfoot load (%)	R	25 ± 1.5	n.s	24.8 ± 1.8	n.s.	17.2 ± 6.9	n.s.	n.s	0.12	0.0001***	1.58	0.0001***	1.5
	L	25 ± 1.2		22.7 ± 6.7		16.8 ± 7.6		n.s.	0.45	0.0001***	2.62	0.001**	0.83
Rearfoot load (%)	R	31.5 ± 1.6	n.s	31.6 ± 1.6	n.s	34.8 ± 3.5	n.s	n.s	0.06	0.006**	1.21	0.01**	1.13
	L	32.3 ± 3		33.4 ± 2.7		35.2 ± 4.1		n.s.	0.57	0.0002***	0.99	0.007**	0.51

Group V1 - women with normally arched feet; Group V2 - women with asymmetrically arched feet (one foot properly arched); Group V3 - women with both feet high-arched; ES – effect size (Cohen d); R – right side; L – left side.

^a^*p* value between right and left side within each group; ^b^*p* value between group V1 and group V2 (the *p* value there is the post-hoc of study groups main effect); ^c^*p* value between group V1 and group V3 (the *p* value there is the post-hoc of study groups main effect); ^d^*p* value between group V2 and group V3 (the *p* value there is the post-hoc of study groups main effect).

Values are expressed as Mean ± SD. *p < 0.05; **p < 0.01; ***p < 0.001.

Analysis of load distribution on forefoot, midfoot and rearfoot showed differences between study groups. In the V3 group (bilateral high-arched), the highest forefoot loading values were noted. Significantly higher values of forefoot load were observed between the V1 and V3 (4.4% for the right side and 4.7% for the left side) and also between V2 and V3 groups (4.4% for the right side and 4.1% for the left side) for both the left and right foot (Table 1). The forefoot load was more noticeable in high-arched feet than in normal arched feet as well in symmetrical and asymmetrical groups. However, no differences were observed between the left and right sides in any of the studied groups (Table 1).

Significant differences in midfoot load were observed between groups V1 and V3 and also between V2 and V3. The percentage differences were 7.8% and 7.6% for the right side and 8.2% and 5.9% for the left side, respectively. Differences between the left and right side were not significant (Table 1).

Significantly higher values of the rearfoot load were observed in the V3 group than those in groups V1 and V2 (Table 1). The percentage difference between the V1-V3 groups was 3.3% for the right foot and 2.9% for the left foot, and between the V2-V3 groups 3.2% and 1.8%, respectively. The effect sizes observed in rearfoot load were weaker than those noted in forefoot load, respectively. Differences in the rearfoot load between the left and right foot were not significant (Table 1).

### Body posture

There were no significant differences in pelvic position variables between study groups (Table 2). Only for pelvic height was there a difference, however, it was not significant. Nonetheless with the high effect size (ES = 0.48), a tendency was observed between groups V2 and V3 groups in which a higher value occurred in the V3 group (Table 2).

**Table 2:** Comparison of body posture variables between study groups.

Outcome Measure	Group V1	Group V2	Group V3	V1–V2^a^	ES	V1–V3^b^	ES	V2-V3^c^	ES
Pelvic torsion (degrees)	4.7 ± 5.3	5.8 ± 3.8	5.3 ± 4.4	n.s.	0.23	n.s	0.12	n.s	0.12
Pelvic obliquity (degrees)	1.7 ± 1.1	1.6 ± 1.2	2.1 ± 1.6	n.s.	0.08	n.s	0.29	n.s	0.35
Pelvic/shoulder obliquity (degrees)	2.1 ± 1.7	2.4 ± 1.4	2.7 ± 2.3	n.s.	0.19	n.s	0.29	n.s	0.15
Pelvic/shoulder rotation (degrees)	3.2 ± 2.7	3.6 ± 3.2	3.8 ± 2	n.s.	0.13	n.s	0.25	n.s	0.07
Thoracic kyphosis (degrees)	33.6 ± 8.7	32.9 ± 8.8	27.8 ± 15.5	n.s.	0.07	n.s	0.46	n.s	0.40
Lumbar lordosis (degrees)	29 ± 8.7	31.4 ± 9.9	29.6 ± 12.1	n.s.	0.25	n.s	0.05	n.s	0.16
Sacral angle (degrees)	24 ± 8	25.5 ± 9.6	24.2 ± 12.1	n.s.	0.16	n.s	0.01	n.s	0.11
Pelvic height difference (mm)	7.6 ± 5.3	6.7 ± 4.9	9.9 ± 7.9	n.s.	0.17	n.s	0.34	n.s	0.48
Scapula distance difference (mm)	7.7 ± 8	5.6 ± 4.4	7.6 ± 6.1	n.s	0.32	n.s	0.01	n.s	0.37

ES – effect size (Cohen d); Group V1 - women with normally arched feet; Group V2 - women with asymmetrically arched feet (one foot properly arched); Group V3 - women with both feet high-arched.

^a^p value between group 1 and group 2 (the p value there is the post-hoc of study groups main effect); ^b^p value between group 1 and group 3 (the p value there is the post-hoc of study groups main effect); ^c^p value between group 2 and group 3 (the p value there is the post-hoc of study groups main effect). Values are expressed as Mean ± SD.

**Figure 2:**
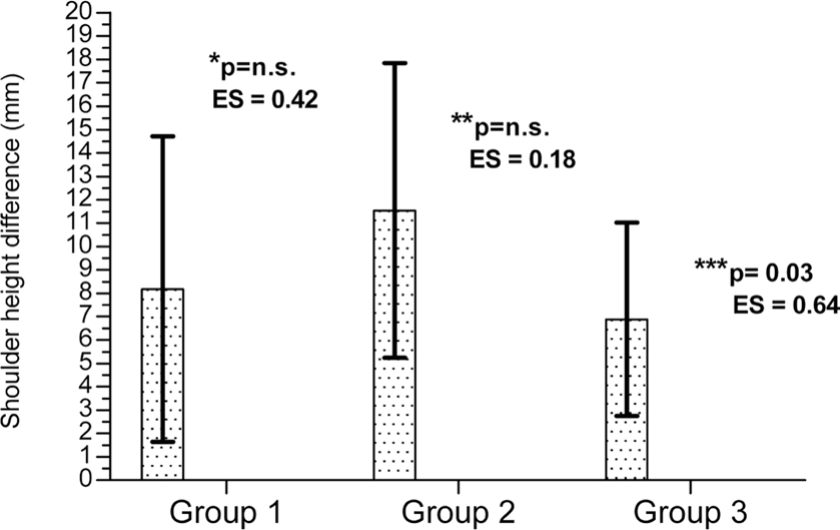
Comparison of shoulder height difference groups.between study. p* - p value between group 1 and group 2 (the p value there is the post-hoc of study groups main effect). p** - p value between group 1 and group 3 (the p value there is the post-hoc of study groups main effect). p*** - p value between group 2 and group 3 (the p value there is the post-hoc of study groups main effect). ES – effect size (Cohen d). Values are expressed as Mean ± SD.

The shoulder height difference was significantly larger in women with asymmetrical arches (V2) than in those with both feet high-arched (V3) (Fig. 2). The difference between the V1 group (both normal feet) and the asymmetric group (V2) showed an insignificant trend with relatively strong effect size (ES = 0.42) (Fig. 2).

**Figure 3:**
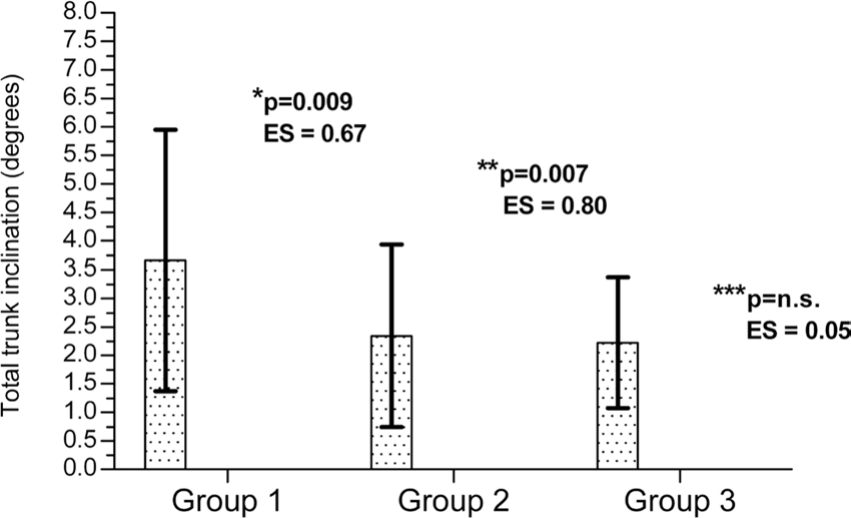
Comparison of total trunk inclination between study groups. p* - p value between group 1 and group 2 (the p value there is the post-hoc of study groups main effect). p** - p value between group 1 and group 3 (the p value there is the post-hoc of study groups main effect). p*** - p value between group 2 and group 3 (the p value there is the post-hoc of study groups main effect). ES – effect size (Cohen d). Values are expressed as Mean ± SD.

The curvature of the spine differentiated the studied groups of women. The highest value of total trunk inclination was found in the V1 group. Significantly lower values of this parameter were noted in both the V2 and V3 groups, but the effect size was stronger between the V1 and V3 groups (ES = 0.8) than between the V1 and V2 groups (ES = 0.67) (Fig. 3). It was notable that the women with high-arched feet and with more pronounced forefoot load also have smaller forward total trunk inclination. No significant differences were found in thoracic kyphosis, lumbar lordosis or sacral angles (Table 2).

## Discussion

The most important observations from this study are that even a slight increase in the arching of the foot in the range that is often not considered as pathological causes visible changes in the distribution of foot loads both between limbs and between the fore- and rearfoot. Slight elevation of the foot’s longitudinal arch may influence the tissues of whole body. The observed increase in forefoot load may result in foot overloading, and what should be underlined is that this may be related to compensatory reactions within the trunk. Our observations may suggest that the consequences of foot high-arching may be present throughout the entire body. Moreover, one-side foot high-arching may be related to asymmetric alignment in the shoulder girdle.

We have observed increased forefoot and rearfoot load in high-arched feet, with the forefoot significantly more loaded. This is a clinically unfavourable phenomenon because forefoot overloading is considered as a pathological pattern of foot load. It was observed that below normal feet, the pressure is slightly higher on the heel than on the forefoot^16^. Our observations were similar to those reported by other authors stating that subjects with high-arched feet have significant reduction in the weight-bearing area, as well as an increase in the load of the forefoot^14,16,33^. Buldt *et al*.^14^ have noted that in subjects with high-arched feet, plantar pressure and force in the forefoot were higher than in subjects with planus feet. Similar results have been noted by Goffar *et al*.^33^. In their study, subjects with high-arched feet had greater force in the medial forefoot region. Burns *et al*.^15^ have observed that about 70% of the subjects with pes cavus reported musculoskeletal foot pain compared to the 23% of individuals with a normal foot type. High-arched feet have a reduced ground contact area, which is rigid and non-shock absorbent^34–36^, and therefore, they are at a greater risk of lower limb overuse injury than in the case of a normal foot^35,37^. It was also suggested that poor distribution of loads in a high-arched foot may induce associated pathology and pain in other areas of the body because of repeated micro-trauma to the legs over an extended period of time^11,15^.

In our study, subjects with high-arched feet who presented increased forefoot load also demonstrated decreased forward trunk inclination in comparison to subjects with normal feet. This decrease in total trunk inclination may be considered as a compensatory reaction when higher loads are shifted to the forefoot. It has been previously suggested that the reduced weight-bearing area under the foot may probably be related to a decrease in afferent proprioceptive stimulation which changes postural corrective reactions^38^. Also, because the plantar muscle together with plantar fascia control the maintenance of the longitudinal foot arch, plantar fascia innervation plays an important role in proprioception, stability and control of foot movements^39^. It was noted by some authors that healthy individuals with different architectural foot types demonstrated some differences in postural control and in subjects with high-arched feet, more proprioceptive stimulation is present in the backfoot^38^ which may explain the decreased trunk inclination among those subjects in our study. Moreover, Cote *et al*.^40^ have suggested that postural stability is affected by foot type under both static and dynamic conditions. They noted that subjects with high-arched feet have better postural control in the posterior and posterio-lateral directions, and concluded that foot type should be considered during the clinical evaluation of balance measurements^40^.

In the group with one-sided elevation of the foot arch, we have observed asymmetrical limb loading, in which greater pressure was directed to the limb with the correct arch of the foot. We have hypothesised that asymmetric limb loading may create an unfavourable tension within the myofascial chains and can lead to alterations in the parts of the body distal from the foot^41^. Shoulder height asymmetry observed in the group with one-sided high-arching of the foot may confirm our suggestions. It was reported that unilateral or asymmetric overpronation of the foot produce a functional difference in lower limb length and a lateral tilt of the pelvis to the side with increased foot pronation^7,42,43^. Also, Duval *et al*.^11^ have demonstrated a transferred effect of unilateral calcaneal eversion up to the thorax. Moreover, Pinto *et al*.^7^ investigated the effects of bilateral and unilateral increases in calcaneal eversion on pelvic alignment and reported an increase in anterior pelvic tilt with both bilateral and unilateral calcaneal eversion and lateral pelvic tilt with unilateral calcaneal eversion.

Most of the studies have reported the influence of low-arched or hyperpronated feet on pelvic and lumbar spine alignment^7,12,42^. There are only a few studies evaluating the influence of foot high-arching and supination on body posture^8,11^. Khamis *et al*.^42^ have evaluated the effect of bilaterally increased calcaneal eversion on pelvic alignment in asymptomatic subjects. They observed that hyperpronation can lead to immediate shank and thigh internal rotation and change in pelvic position^42^. On the other hand, Duval *et al*.^11^ investigated whether foot supination (measured as calcaneal inversion) induced posterior pelvic tilt and decreased lumbar lordosis. They showed that there were no alterations in pelvic tilt or lumbar lordosis, and suggested that the effects of calcaneal movement were too small to be detected in the pelvis and lower back^11^. Betsch *et al*.^8^ also did not find a correlation between foot position and changes in spinal posture. The results from our study are in agreement with the observations of Duval *et al*.^11^ and Betsch *et al*.^8^. We also did not observe any differences in pelvic and lumbar position between subjects with high-arched and normal feet.

It is important that alterations in pelvic or trunk posture observed by some authors were a result of the immediate change of foot position by wedges or special sandals^12,42^. Additionally, it was suggested that the soft tissues between the foot and pelvis in the biokinematic chain have some elasticity and may adapt before the movement of one segment affects the other^11,44^. Therefore, those observations should not be transferred to the condition in which foot posture alterations are permanent and not induced only for the purpose of research. Tateuchi *et al*.^12^ have observed that patients with foot pathologies may alter their posture to adapt to the foot deformity. As was underlined by Mueller *et al*.^45^, long-term tissue adaptations may allow the occurrence of greater postural changes. In our study, the changes in foot posture were not induced during the experiment, but were habitual changes in foot arch. Consequently, body tissues adapted in the case of some parts, and therefore, we probably did not note differences in pelvis inclination or spinal angle. Some studies demonstrated that dysfunction may occur beyond initially affected area. Other muscles could begin to compensate for the dysfunction which might overload the compensatory muscles. It could cause in decreasing functional range of motion and causing pain or dysfunction in this muscle group^46,47^.

There are some study limitations which should be addressed. All measurements were performed in a static position, therefore, the results should not be fully extrapolated to dynamic conditions. In the future, it would be interesting to conduct measurements, e.g. during gait, which may show how excessive foot arching affects load distribution and body posture in dynamic conditions. Moreover, further research including patients with different foot pathologies may provide a better understanding of forces transmitted via kinematic chains from the foot to the hip, pelvis and thorax.

## Conclusions

Our study has shown that a slight increase in longitudinal arch of the foot, even within the range that is often not considered as pathological, causes visible changes in the distribution of feet loads both between limbs and between the forefoot and rearfoot. High-arching of the feet may also influence the whole body. Moreover, asymmetrical high-arching of the feet resulted in asymmetry of lower limb load and in the shoulder girdle, of height. We have suggested that any alteration of the foot arch may be harmful to body tissues and should not be considered as correct (within the limits of the norm). Due to the fact that slight increases in longitudinal arch of the foot are very common, they should be considered as a foot defect, and appropriate corrective exercises should be used to prevent forefoot overload and alterations in body posture.

The clinical implications of this study have indicated that alignment of the foot should be considered as a contributing factor in diagnosis of all musculoskeletal dysfunctions within the lower limbs, the pelvis, spine and thorax. We believe that a more comprehensive understanding of how each part of the biokinematic chains influence each other will provide a better basis for assessment and treatment of body malalignment.

## Data Availability

All data generated or analysed during this study are included in this published article.
